# Polysaccharide-Based Edible Gels as Functional Ingredients: Characterization, Applicability, and Human Health Benefits

**DOI:** 10.3390/gels8080524

**Published:** 2022-08-21

**Authors:** Mihaela Stefana Pascuta, Rodica-Anita Varvara, Bernadette-Emőke Teleky, Katalin Szabo, Diana Plamada, Silvia-Amalia Nemeş, Laura Mitrea, Gheorghe Adrian Martău, Călina Ciont, Lavinia Florina Călinoiu, Gabriel Barta, Dan Cristian Vodnar

**Affiliations:** 1 Life Science Institute, University of Agricultural Sciences and Veterinary Medicine, 400372 Cluj-Napoca, Romania; 2 Department of Food Science, Faculty of Food Science and Technology, University of Agricultural Sciences and Veterinary Medicine, Calea Mănăștur 3-5, 400372 Cluj-Napoca, Romania

**Keywords:** polysaccharides, edible gels, functional ingredients, food applicability, health benefits

## Abstract

Nowadays, edible materials such as polysaccharides have gained attention due to their valuable attributes, especially gelling property. Polysaccharide-based edible gels (PEGs) can be classified as (i) hydrogels, (ii) oleogels and bigels, (iii) and aerogels, cryogels and xerogels, respectively. PEGs have different characteristics and benefits depending on the functional groups of polysaccharide chains (e.g., carboxylic, sulphonic, amino, methoxyl) and on the preparation method. However, PEGs are found in the incipient phase of research and most studies are related to their preparation, characterization, sustainable raw materials, and applicability. Furthermore, all these aspects are treated separately for each class of PEG, without offering an overview of those already obtained PEGs. The novelty of this manuscript is to offer an overview of the classification, definition, formulation, and characterization of PEGs. Furthermore, the applicability of PEGs in the food sector (e.g., food packaging, improving food profile agent, delivery systems) and in the medical/pharmaceutical sector is also critically discussed. Ultimately, the correlation between PEG consumption and polysaccharides properties for human health (e.g., intestinal microecology, “*bridge effect*” in obesity, gut microbiota) are critically discussed for the first time. Bigels may be valuable for use as ink for 3D food printing in personalized diets for human health treatment. PEGs have a significant role in developing smart materials as both ingredients and coatings and methods, and techniques for exploring PEGs are essential. PEGs as carriers of bioactive compounds have a demonstrated effect on obesity. All the physical, chemical, and biological interactions among PEGs and other organic and inorganic structures should be investigated.

## 1. Introduction

Due to their versatile properties, polysaccharides-based edible gels (PEGs) are intensively studied. Polysaccharides are biopolymers that belong to the class of carbohydrates. They are formed by polymeric chains of oligosaccharides linked by glycosidic bonds. They are highly abundant in nature, with low-cost production, non-toxic, biocompatible, biodegradable, bioactive and water-soluble [1,2,3]. Defining gels in detail is complex, but the term “gel” is commonly associated with highly hydrated structures containing two components in varying proportions, inspired by the “peel/skin” of fruits and vegetable [4]. More exactly, gels are semi-solid systems comprising a solid three-dimensional (3D) network (the ‘gelator’) that traps the liquid phase [5].

**Figure 1:**
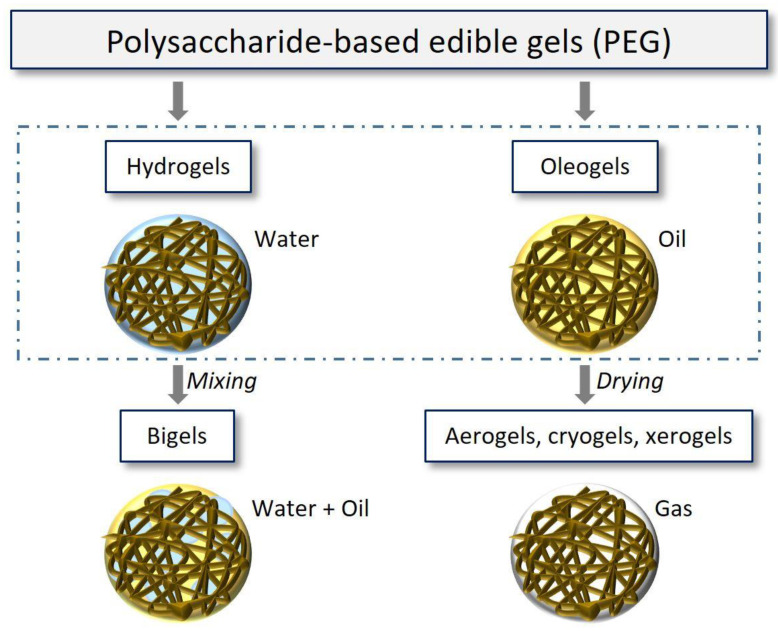
Classification, structure, and formulation of polysaccharide-based edible gels.

PEGs can be classified into (i) hydrogels; (ii) oleogels (or organogels) and bigels, and (iii) aerogels, cryogels, and xerogels, respectively (Figure 1). Hydrogels are hydrophilic molecules, with water as the predominant phase and one or more composite biopolymers. Oleogels have lipids as the predominant entrapped in oleogelators (emulsifiers or biopolymers) as a three-dimensional (3D) network [6]. Bigels are mixtures of both hydrogels and oleogels that contains both water and oil as the predominant phase. Despite a lot of effort being spent on bigel characteristics, such as their microstructure and mechanical properties, this field is still in the incipient phase of development [5]. Aerogels are formed from a 3D biopolymer chain that retains the network of the wet state (usual hydrogels) while drying. The solvent from the wet state is replaced by a gas [3]. Aerogels can be classified on the basis of their method of preparation, especially drying method. These can be described as aerogels (supercritical drying), cryogels (freeze-drying and freeze-thawing), and xerogels (vacuum drying) [7]. Thus, each class is characterized by specific properties. For example, xerogels are denser than aerogels [8], while cryogels has larger macropores and lower specific surface area than aerogels [9].

PEGs have been recognized as an alternative and beneficial solution with numerous applications due to their natural, low cost, and biodegradability, having an advantageous position not only in the food sector but also in the medical/pharmaceutical sector [10]. In the food sector, PEGs have found applicability for improving food profile and in the food packaging industry. PEGs can form edible coatings that cover foods or control the release of entrapped active compounds into the food [11,12]. PEGs can be used as a delivery system for unstable and easily degradable bioactive compounds (e.g., polyphenols, vitamins, carotenoids, probiotics) that imprint flavor, aroma, and colour [13,14,15,16]. Furthermore, PEGs facilitate the adsorption of such compounds into the intestines [17,18]. With respect to above-mentioned applications hydrogels was critically discussed and considering their immense potential, further studies have an ample scope [19]. Furthermore, oleogels represent promising alternatives to solid fats for food application and their recent advances in food application was critically discuses [20]. In the medical/pharmaceutical sector, PEGs are mainly used as a drug delivery systems [1] and for wound healing [21]. For example, the application of aerogels in medical sector were recently revised [22,23,24].

PEGs have shown promising benefits for human health, mainly due to polysaccharide content. Their positive benefits are related to the gut microbiota, intestinal microecology, and the “*bridge effect*” in obesity. For instance, dietary PEGs modulate the gut microbial population by reducing pathogen development, controlling probiotics, and increasing the host-microbial interaction [25,26,27]. Obesity can be managed by using PEGs due to their properties to deliver both hydrophobic and hydrophilic nutraceuticals in a controlled manner. PEGs, such as hydrogels, can protect bioactive compounds through the gastro-intestinal tract [18,19,28]. The past five years have seen tremendous report research on PEG development and its valuable applicability. Since this domain is found in the incipient phase, the focus is mainly on sustainable biopolymers as raw materials, preparation techniques, and characterization of PEGs [3] for a better understanding of PEG identities. For instance, pectin hydrogels, aerogels, cryogels, and xerogels were recently obtained and the influence of the drying method on their structural and release properties was evaluated [9]. Cellulose-based aerogels, cryogels, and xerogels were designed as hierarchical porous structures [29]. Furthermore, despite PEGs using common raw materials as 3D networks (e.g., polysaccharides), each class of PEG is usually individually treated for its potential and applicability. To the best of our knowledge, there is no manuscript that gives an overview of the whole developed PEG. Therefore, the novelty of the present paper is to critically review the latest literature on PEGs with respect to their classification, definition, formulation, characterization, and specific properties. Furthermore, functional and health-related attributes and industrial applications are critically discussed to better understand their future directions and potential sustainable production.

## 2. Polysaccharide-Based Edible Gels: Formulation, Properties, and Characterization

### 2.1. Hydrogels

#### 2.1.1. Hydrogels Definition and Formulation

The term “hydrogel” denotes a network of hydrophilic molecules or polymers that can swell when exposed to water and hold a significant amount of water inside their bulk. The generation of hydrogel particles based on polysaccharides can be accomplished using various methods. The formation of spherical droplets/particles utilising a combination of the bioactive component and polymeric excipients is the foundation of numerous techniques. The shape of polysaccharide particles, which determines the size and size distribution of the resulting microparticles—the two main variables determining the release of bioactive compounds is essential for the development of such polysaccharide-based hydrogel particles [2].

The methods used to create monodispersed particles from polysaccharide droplets can generally be categorised as follows:

(1) Gaseous droplets’ development and subsequent fall into a gelling medium.
(2) The development of droplets in a liquid phase that is immiscible with the polymeric solution; in this situation, mixing results in an emulsion.

The essential parameters that influence the size and shape of the liquid droplets in both techniques are the viscosity of each phase, the surface tension of the polysaccharide solution compared to the surrounding medium (gas/air or liquid), and the dynamic interactions of the droplets with the matrix fluid (laminar or turbulent flow). Surface tension at the liquid-air interface, or case (1), when liquid is forced through a nozzle at a constant flow rate, is crucial. In the second instance (2), the liquid is broken down into droplets in an immiscible fluid system, and surfactants are typically used to regulate the interfacial tension between the dispersed and continuous phases [22].

#### 2.1.2. Hydrogels Properties and Characterisation

1. *Mechanical and rheological properties*: Polysaccharides possess a wide range of physical, chemical, and biological properties, which contribute to their high biocompatibility, bioactivity, and degradability. The presence of freely available functional groups such as carboxyl (–COOH), amino (–NH3), hydroxyl (–OH), and other hydrophilic groups allows a series of chemical modifications which suit the desired applications. In addition, oxidation of some polysaccharides by periodates under acidic conditions leads to the formation of 2,3-dialdehyde polysaccharides. These compounds can form cross-links with protein side chains or with amino groups present in chitosan, for example. However, alginic acid, a polysaccharide from brown seaweeds, has been used to cross-link collagen films, while di- and polycarboxylic acids, such as citric acid, may be used as cross-linking agents for polysaccharide films [30]. The gelation process is defined as the gradual cross-linking of polymer chains inside the reaction system to form a single enormous molecule of “infinite” size [31]. As mentioned above, polysaccharides have many functional groups, making it possible to cross-link them physically or chemically to create a hydrogels [32,33]. Physical cross-linking of polysaccharide polymers typically involves weak interaction forces such as H-bonding/Van der Waals interactions/hydrophobic forces and molecular entanglements [34], whereas chemical gelling frequently entails covalent bonding between polysaccharide polymer(s) and the corresponding cross-linker, including condensation reactions, enzymatic disulfide cross-linking, click chemistry, polymerisation, etc. [35]. For example, a cellulose-based hydrogel created through chemical cross-linking with cationic polyelectrolytes was described by Yang et al. (2021). The ionic sites are mainly –COO– (in carboxymethylcelluloses, pectins and alginates), –NH_3_ (for chitosan in acidic media) and –SO_3_ (in carrageenan). In the case of polyelectrolytes with –COO– and –NH_3_ functions, the net charge will depend strongly on the pH in connection with the dissociation equilibrium. The electrostatic potential of the polyelectrolyte grows progressively as the degree of polymerization increases and goes to a limit as soon as the number of charges (or degree of polymerization) is larger than 15. Then, the thermodynamic properties become independent of the molecular weight [36]. The hydrogels produced had high tensile strength (21–51 kPa), a high tensile strain (899–1047%), and good compressive properties, which made them adaptable to repeated joint movements without coming loose [37].
2. *Moulding time*. Rapid ionic gelation is one example of how quickly hydrogels can be physically cross-linked. For instance, hydrogen bonds, host-guest chemistry, hydrophobic interaction, stacking interaction, and coordination bonds are a few more physical cross-linking techniques that can be finished in a matter of hours [38]. Chemical cross-linking, however, typically requires more time. Hydrogels formed by chemical cross-linking show greater stability, longer durability, and higher mechanical properties (tensile, shear, bending, etc.), compared with physically cross-linked hydrogels [39]. Juho Lee et al. (2019) created a hydrogel-forming based on in situ alginates that quickly absorbed fluids and gelled them [40]. Rapid in situ gelation properties make the hydrogel more transportable and easier to use in the pharmaceutical or healthcare sectors.
3. *Swelling and moisturization*. The degree of cross-linking has a direct impact on how well a hydrogel swells and retains moisture. In general, the swelling performance degrades as cross-linking increases. The cross-linking immediately affected how well a hydrogel swells and retains moisture. Generally, the swelling performance degrades as cross-linking increases [41,42]. By cross-linking the low-methoxyl pectin with calcium ions, Lou et al. (2011) created a hydrogel that could absorb tissue fluid and keep the wound wet. The findings also indicated that the formulation composition’s ratio affects the qualities of swelling and water retention [42]. For instance, a sodium alginate-based hydrogel was created with effective self-healing (80% mechanical recovery in 6 h), high tensile strength (0.109 MPa), and ultra stretchability, which are thought to be desired qualities and superior to previously described tough and self-healing hydrogels for wound dressing applications [43]. By combining covalent and physical cross-linking techniques, Suflet et al. (2021) created stable chitosan-based hydrogels with a relatively high swelling rate and low elastic modulus (3–30 kPa) [44].
4. *Biological functions*. Numerous functional groups, such as carboxyl and hydroxyl groups on the backbones of polysaccharide polymers, enable adaptable alterations of the hydrogel to mix with various medications and then release them in response to diverse stimuli [45]. Deng et al. (2021) also described a sodium alginate-based hydrogel with metal-organic frameworks that resembled semiconductors and contained noble metal nanoparticles. The composited hydrogels dramatically hastened wound healing and exhibited extraordinary bactericidal activity against both *E. coli* and *S. aureus* through forming reactive oxygen species [46]. According to Bystroová et al. (2018) and Afewerki et al. (2019), natural bio-polysaccharide macromolecules can interact with proteins to generate glycoproteins that imitate the extracellular matrix [47,48]. These glycoproteins are essential cues for controlling cellular processes and directing tissue repair and regeneration. Human epidermal keratinocyte proliferation, viability, adhesion, and spreading were all significantly improved by the biologically active hydrogel made of alginate and gelation compared to the control. This hydrogel also showed few adverse effects on the samples of the restored human epidermis in the in vitro skin irritation test method [48]. According to reports, the polysaccharide molecules produced when the hydrogels degrade are involved in various immune processes, enabling pro- and counter-inflammation and wound healing via the immunomodulation [49]. According to Zhu et al. (2019), an alginate-gelatin-hydrogel combination stimulated the proliferation and migration of fibroblasts in a macrophages/fibroblasts co-culture system. In addition, when compared to the equivalent fibroblasts or macrophages monocultures, the expression of inflammatory cytokines and chemokines was improved [39,50].

### 2.2. Oleogels and Bigels

#### 2.2.1. Oleogels and Bigels Definition and Formulation

**Figure 2:**
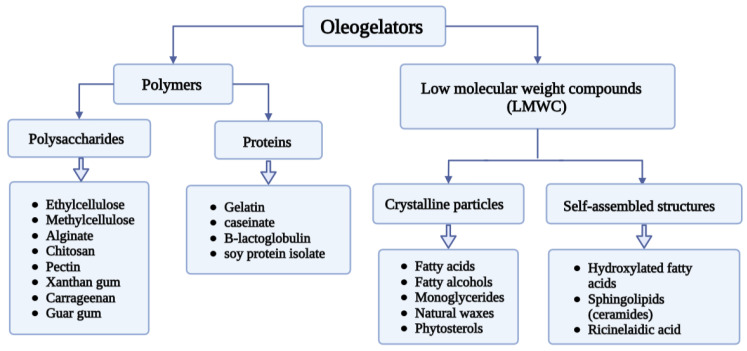
Most widely used edible oleogelators (image created using the BioRender application; https://app.biorender.com).

Oleogels are gels in which the continuous liquid phase is represented by vegetable oils. Oleogels are solid-like systems where the constant liquid phase is entrapped or immobilised in a 3D network. To create a semisolid or solid-like network structure, alternative structuring agents are needed in the process, precisely oleogelators. According to Li et al. (2022), oleogelators can be represented by monoacylglycerols, diacylglycerols, fatty acids/alcohols, sterols, waxes, and ethyl cellulose, which are the well-known and widely used edible oleogelators (Figure 2). The gelling ability of the oleogelator mainly depends on the balance between their solubility and insolubility in the oil phase, as well as on the self-assembly and self-organisation into higher ordered structures (e.g., liquid crystals, crystal lattice, bilayers, micelles, fibrils) [20].

Oleogels (or organogels) formation can be divided into two strategies: (i) direct approaches, through a short dispersion of the melted oleogelators into an oil medium followed by a cooling step, which leads to a self-supporting gel, and (ii) indirect approaches. The indirect approaches usually involve preliminary preparation of hydrocolloids by using biopolymeric oleogelators of hydrophilic nature. Hydrophilic polymers such as chitin, β-lactoglobulin, soy protein, gelatin, and triterpene saponin, have been reported to develop oleogels through several indirect methods such as emulsion template, foam template, hydrogel template, or solvent exchange [51,52]. According to Li et al. (2022), the gelling ability of these oleogelators mainly depends on the balance between their solubility and insolubility in the oil phase, as well as on the self-assembly and self-organisation into higher ordered structures (e.g., liquid crystals, crystal lattice, bilayers, micelles, fibrils) [20]. Bigels are another type of PEG. Bigels are novel biphasic systems with different polarity phases, having stable structures. Compared to oleogels and hydrogels, bigels possess both phases’ advantages and show better properties than either of the individual gel components due to the strong interaction produced by dual gelators [53]. Recently, Martín-Illana et al. (2022) critically reviewed bigel manufacture together with sustainable raw materials for hydrogels and oleogels, as well as the used solvents for bigel preparation [54].

#### 2.2.2. Oleogels and Bigels Properties and Characterization

The chemical composition of the oil phase and the oleogelators, their molecular structure, and the intermolecular interactions between them, significantly impact the formation, microstructure, and characteristics of oleogels. Depending on the molecular weight of the oleogelators, the oleogels can be categorised as low molecular weight oleogels or polymeric oleogels. Thus far, ethyl cellulose (EC) is known as the only polymeric oleogelator to structure oil through direct addition. In this regard, a previous comprehensive review about the utilization of EC in food applications illustrates in detail the production, structure, and properties of pure EC, as well as the mechanism of gelation and the parameters affecting the final gel properties [55]. The primary characteristic of the oleogels is that they can be developed with zero-trans and reduced saturated fats, making them a healthier lipid compared to conventional solid fats [56].

Regarding bigels, one of the essential characteristics, besides spreadability, easy preparation, moisturising effects, excellent mechanical and storage stability at room temperature, and improved absorption of drugs through the skin, is the combined delivery capacity of hydrophilic and hydrophobic agents at the same time. A recent study regarding the characterisation of hydrogel-oleogel biphasic systems offers crucial structural information about the bigels as affected by different oleogelators, which could be helpful in the development of novel functional food products [53].

### 2.3. Aerogels, Cryogels and Xerogels

#### 2.3.1. Aerogels, Cryogels and Xerogels Definition and Formulation

Aerogels, cryogels and xerogels, respectively, represent three common categories of lightweight materials with unique properties. These are obtained using the same principle: wet-gel (or wet polymer network, usually hydrogel) is dried using advanced techniques and the liquid phase is replaced by gas. The used drying method define each category of materials, as well as their properties, as follows in the next rows.

Aerogels are 3D, ultralight porous materials composed of polymer chains that have retained the nano-porous networks of the wet state while drying [57]. Aerogels are dry open pores nanostructured (mesoporous with small macropores) materials with >90% porosity and >100 m^2^/g surface area; despite no formal agreement existing [7]. To form aerogels, the liquid component of the gel must be extracted through supercritical drying. This method conducts to the highest pore volume and the most extensive texture range [3,7,58]. Compared to conventional evaporation, supercritical drying allows the liquid to dry slowly meanwhile integrating the gas in the solid matrix of the gel without producing a collapse from the capillary action. The outcome is a gel solid structure [59,60]. Polysaccharide-based aerogels have been reviewed recently with respect to their classification [61], characterization methods, and state of the art, respectively [22]. Benefits and drawbacks of drying methods and raw materials for aerogels production were already discussed [23].

**Figure 3:**
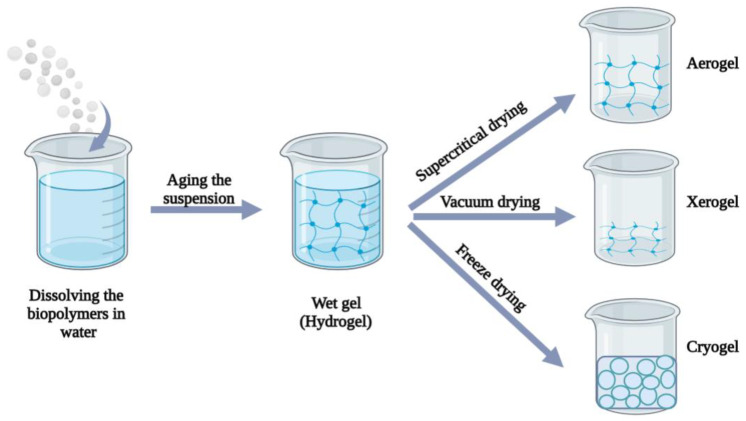
Schematic illustration of the preparation of aerogels, xerogels and cryogels from hydrogels (image created using the BioRender application; https://app.biorender.com).

Xerogels are obtained by low vacuum drying (or ambient drying), where the solvent (e.g., water, alcohols) evaporates. The physical properties of the solvent medium and the liquid-vapour interface influence the properties of xerogels [59]. Xerogels becomes denser than aerogels due to the formation of smaller pores (1–10 nm). Moreover, xerogels maintain porosity values between 15–50% alongside a lower surface area [8]. Their appearance is similar to a thin film without noticeable pores [59]. In order to develop cryogels, both freeze drying (lyophilization) and freeze-thawing methods were used. Freeze drying is a process that involves freezing the gel, lowering the pressure, and then removing the frozen solvent by sublimation. Freeze-drying significantly damages the gel’s initial pore structure, leading to the formation of fissures. Additionally, this method modified the shape of the pores, leading to thicker pore walls than the aerogels [23]. Recently, Chartier et al. (2022) obtained cryogels by freeze-drying, and aerogels by supercritical CO_2_ drying as chitosan-based porous materials. Cryogels possessed a lower density, a lower specific area, by a higher porosity in comparison to aerogels [62]. The freeze-thawing method generally involves preparing the wet-gel, freezing of wet-gel, and thawing the frozen system, respectively [63]. For example, cyclic freeze-thawing improved mechanical strength of cellulose nanofibril-based cryogels. These exhibited excellent wet strength and shape retention without cracking under hand squeezing [59]. Figure 3 shows the preparation method of aerogels, xerogels, and cryogels, respectively.

#### 2.3.2. Aerogels, Cryogels and Xerogels Properties and Characterization

Aerogels are characterised by high porosity, very low density, great active surface area and shallow thermal conductivity [60]. These properties make aerogels unique materials, attractive for quite a few applications varying from thermal insulation (being nonconductor materials) to biomedical and pharmaceutical purposes [64]. Aerogels (99.8% air) are composed of air pockets and spherical particles with an average size of 2–5 nm, which are bonded together into clusters or groups [57]. These groups form the 3D porous structure, having the pores dimension under 100 nm. The lack of solid material makes aerogels nearly weightless [7]. Although, a 2 g piece of aerogel can sustain a 2.5 kg brick [3]. Generally, xerogels are exposed to the higher collapse of the solid gel structure than aerogels, resulting in lower porosity but a superior bulk density [59,65]. Due to these characteristics, in most cases, the mechanical properties of xerogels are greater than aerogels [58]. The xerogels applications are related to phage therapy, enzyme immobilization or topical purposes [66]. Cryogels are characterized by a low value of the specific surface area, highly-interconnected microporous networks (which can vary from a few micrometres to hundreds of micrometres in diameter), and excellent mechanical properties [58]. Especially the microporous structure and other characteristics make cryogels appropriate for developing a bioscaffold platform with tissue engineering applications and cellular therapies [7,58]. Aerogels may be characterized by determining (i) structural properties (e.g., bulk density, porosity), (ii) morphological properties (e.g., scanning electron mycroscopy, SEM); (iii) texture properties (e.g., specific surface area, pore volume, pore size distribution) [22]. Table 1 shows the main characteristics of aerogels, cryogels and xerogels, together with few examples of already produced PEGs.

## 3. Applicability of Polysaccharide-Based Edible Gels

**Figure 4:**
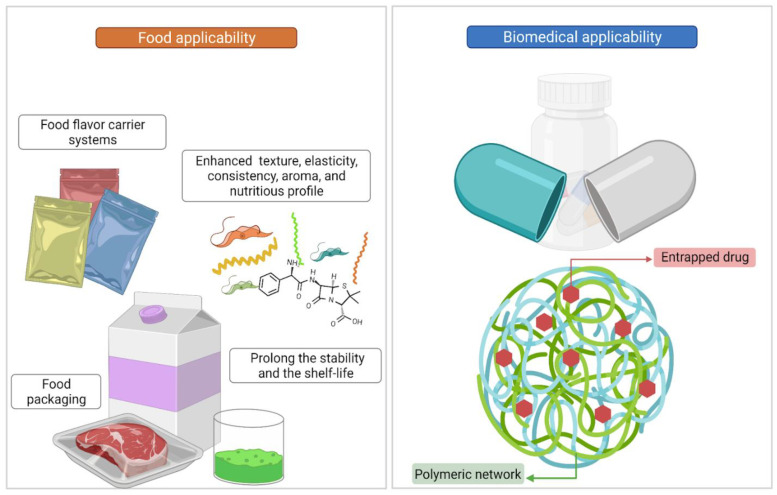
PEG applications in the food and biomedical sector (image created using the BioRender application; https://app.biorender.com).

Polysaccharides are biomolecules found in various natural sources, and they have great potential applicability in the biotechnology and food industries as PEGs. The relevance of PEGs in the food industry is based on its characteristics of enhancing food structural properties (e.g., texture, elasticity, and consistency), increasing the nutritional profile, extending food shelf life, and improving the food-packaging properties. On the other hand, the biomedical application of PEGs comprises innovations in the drug delivery system, the development of novel biomaterials required for medical devices, and the development of medical biosensors. Some of the PEG applications in the food and biomedical industry are shown in Figure 4 and detailed in the following lines.

### 3.1. PEGs in the Food Sector

#### 3.1.1. Agents for Improving Food Profile

With the growing consumers’ demand for healthy and appealing foods, the use of PEGs gained popularity in the food sector for developing products with enhanced properties in terms of texture, elasticity, consistency, aroma, and nutritional profile. Organic gels such as PEGs are much more exploited lately as they play a significant role in formulating food flavour carrier systems. At the same time, they have multiple benefits for food applications considering that they prolong the stability and the shelf-life of the products incorporating them [19,70].

The organic gels used for food designing, preparation, and enhancement of structural stability are edible biopolymers which are declared as Generally Recognized As Safe (GRAS) by the Food and Drug Administration (FDA) [19,71]. Biopolymers such as sodium alginate, chitosan, agarose, starch, pectin, agar-agar, or hyaluronic acid create 3D structures that facilitate the integration of some bioactive molecules, water, or oils in the mesh of the network, giving the FERM structure of gels that can be shaped into a variety of sizes and forms, the system that is highly desired in both traditional and modern gastronomy preparations [72,73,74,75]. Nonetheless, some biopolymers imprint the gel structure to the food matrices by stabilizing the internal forces in internal phase emulsions, attributing at the same time improved rheological properties in terms of viscosity, shear strain, or storage modulus [12,76,77,78].

PEGs that are met in food recipes are mainly derived from microbial entities (e.g., xanthan gum produced by *Xanthamonas* sp., succinoglycan produced by *Agrobacterium tumefaciens*, glucan produced by *Leuconostoc dextranicum*, etc.), from seaweeds or marine macroalgae (e.g., fucoidans extracted from brown algae, carrageenan extracted from red seaweeds, ulvans extracted from green macroalgae), from plants (e.g., pectin from citrus peels, cellulose from pruning waste, starch from potatoes or cassava, etc.), or even from animals (e.g., chitosan from shell crustacean, glycogen and gelatin from animal tissues, etc.) [79,80,81,82]. These structures are very efficient in the 3D constructions of artistic plates and delivering nutrient-rich food products [83,84,85]. In addition, the polysaccharide complexes as stabilizers in different Pickering emulsions targeting to adjust protein or lipid-based formations [86]. Furthermore, the linkages among the polysaccharide and proteins or fats increase the hardness, adhesiveness, and chewiness, especially in gels based on alginate, maltodextrin, pectin, chitosan, or starch [87,88]. In protein-polysaccharide structures, electrostatic interactions appear to lead to the coacervate formation, further contributing to the particular properties of the protein-polysaccharide gels [17]. Nonetheless, protein-polysaccharides-based gels are of very much interest lately as they are effective in delivering unstable and easily degradable bioactive compounds (for example, polyphenols, flavonoids, carotenoids, vitamins, probiotics, etc.) that particular imprint flavor, aroma, and colour to the food matrix, and facilitate their efficient absorption at the intestine level [17,18]. For instance, carotenoid-enriched lipid droplets had higher bioaccessibility when incorporated into starch-based hydrogel. Hydrogel beads modulated gastointestinal fate of carotenoids due to their ability to inhibit excessive droplet flocculation in the stomach and small intestine and allowed easy access of lipase to the lipid droplet surfaces [5].

Hydrogels may impact the texture properties of food due to their texture properties (elasticity, hardness, chewiness, fracture, etc.). As a future perspective, food with a reduced caloric content may occur by replacing meat or starch with hydrogels to that of excellent texture characteristics, or of low oil content. Furthermore, pancakes with reduced caloric density and prepared at higher temperature than the boiling point of water were produced by using a temperature insensitive, food-grade, mixed agar-methylcellulose hydrogel [89]. Aerogels may enhance taste and flavour perception of food due to the proportion of air inclusion in the gel matrix. The high diffusion rate of tastants when air is included contributes to an increased delivery and perception of saltiness, sweetness, and flavor. Therefore, aerogels may reduce energy and salt intake through the diet [90].

For instance, future perspectives linked to the applicability of oleogels in food serve as delivery systems and other roles. Given the wide variety of vegetable oils, the large array of oleogelators and the multiple methods of oleogelation, tremendous assortments of oleogels can be developed. According to Puscas et al. (2020), the applicability of oleogels in food products primarily depends on their properties, such as texture, spreadability and sensory properties. It is determined using critical gelling concentration and solid fat content [91]. This study reflects the up-to-date applications of oleogels in food products and debates the properties and the techno-functionality of reformulated food products compared to the reference products. The edible applications of oleogels are concentrated in five main categories: spreads; confectionery such as chocolate, pralines or fillings; pastry such as cookies, biscuit cakes and baked goods; meat products including Frankfurters, sausages, meat patties or Pâtés; and dairy products such as cream cheese or ice-cream.

Oleogels can serve as a delivery system for a controlled/delayed release of fat-soluble nutraceuticals and pharmaceuticals. A recent study regarding oleogel-based methods for delivering bioactive compounds summarizes the development, production, characterization, and applications of oleogels to transfer functional molecules in the foods [92]. According to Pinto et al. (2021), the structure of oleogels constitutes a suitable matrix for the delivery of bioactive molecules by controlling their release and protecting the integrity of bioactive compounds against oxidation or loss of functionality at the same time. Most of the studies, though, are focused on lipid-soluble bioactive compounds such as curcuminoids, carotenoids, coenzyme Q10, docosahexaenoic acid, conjugated linoleic acid or plant sterols due to the lipophilic nature of oleogels [91,92].

With oleogels’ applicability, bigels can serve as reliable vehicles for bioactive molecules regardless of their hydrophilic or hydrophobic nature. Previous work on the printability and rheological properties of bigel inks showed that the 80% oleogel fraction group was an ideal material for the 3D printing [93]. 3D food printing is an excellent tool for personalised nutrition about particular dietary necessities (e.g., obesity). It can be an ideal instrument to prevent different non-communicable diseases (e.g., gut microbiota, intestinal microecology) through improved functional food products containing bioactive compounds (e.g., antioxidants, phytonutrients, proteins, and probiotics) [83,94,95,96]. The compatibility between bigels, serving as vehicles for both hydrophilic and lipophilic bioactive molecules, and personalized 3D food printing might have impactful results regarding the prevention or the treatment of individual needs in humans. However, in vitro and in vivo studies would be desirable to investigate the bioavailability and bioaccessibility of the active compounds during digestion.

#### 3.1.2. Food Packaging

In addition to acting as valuable food ingredients for functional foods production, polysaccharide structures play an essential role in developing packaging materials or active films for coating different food products to extend their shelf-life [12,97]. For example, polysaccharide-based aerogels are new coating materials with decreased weight and density but increased mechanical strength, high porosity, and generous surface area. In the same line, aerogels are used as smart packaging materials that facilitate the controlled release of active compounds into food matrices, exerting antibacterial and antifungal properties, a fact that makes them available as drug delivery systems as well [90]. Aerogels consisting of cellulose, hemicelluloses, marine polysaccharides, or starch act also as efficient absorbers of moisture and can be mainly used in the packaging of meat products to avoid water condensation and to reduce the water activity, factors that are responsible for the microbial growth [98,99]. Moreover, aerogels based on pectin have the potential for insulated food packaging applications to limit the heat transfer and keep the temperature at constant values within acceptable minor variations. Additionally, pectin-based aerogels enriched with active nanoparticles such as TiO_2_ have both increased antibacterial potential, moderate mechanical properties and lower thermal conductivity than air [67].

Edible coating materials can be created starting from polysaccharides of organic nature, replacing at the same time the traditional packaging materials with potentially negative environmental impacts, such as plastic materials, metals, glass, and paperboards. PEGs obtained from carrageenan can be used as coating membranes for different solid foods (e.g., fresh-cut foods), giving good stability to the food product by reducing the microbial activity [97,100]. Chitosan and alginate are two largely exploited polysaccharides producing edible coating materials with bioactive properties. Chitosan, for example, creates form gels based on its inter- and intra-molecular cross-linking protonated amino groups, which makes chitosan more appropriate for thermo-stable films with decreased solubility, and more compacted and dense coating materials with antibacterial properties available for food sector [101]. Alginate is mostly used as an encapsulation matrix and in controlled-released systems. It is very effective in protecting the bioactive compounds against acidic conditions, especially from gastric fluid activity. Alginate also forms gels that exert antimicrobial properties against particular pathogenic strains belonging to *Listeria*, *Escherichia*, *Salmonella*, *Pseudomonas*, etc. [102,103]. Moreover, alginate or agar-agar-based gels enriched with polyphenols extracted from different aerial plants (e.g., *Larrea nitida*) form biofilms with improved barrier and mechanical performance, while exerting antioxidant and antimicrobial activities when applied directly to the fresh fruits and vegetable surfaces [11,104].

Moreover, PEGs play a significant role in developing smart materials both as ingredients and as edible, biodegradable, and safe coating materials, and new methods and techniques for providing and exploring them are necessary.

### 3.2. PEGs in the Medical and Pharmaceutical Sector

The abundance of natural polysaccharides, their low-cost processing, biocompatibility, biodegradability, non-toxicity, water solubility, and bioactivity make them the most common and promising biomaterials in the nanomedicine [1,2,3]. Polysaccharides and their derivatives are now being studied for their possible utility as nanoparticles, such as nanogels or micelles in drug delivery systems, due to their biodegradability and non-toxic end products [1,3,105,106]. The primary goal of a drug delivery system is to facilitate pharmaceutical agents into the systemic circulation while controlling the pharmacokinetics, pharmacodynamics, non-immunogenicity, non-specific toxicity, and bio-recognition of the target site to attain the required pharmacological activity [1]. As a result, many polysaccharide structures in the form of aerogels, hydrogels, and oleogels are being designed for diverse applications in the medical and pharmaceutical industry, such as drug delivery [107,108], biomedical devices [109,110], biosensing, and antibacterial [23,111].

PEGs, aicrocapsules, microspheres, nanoparticles, and nanocapsules, are among the most commonly utilised drug delivery systems [108]. PEGs created for drug delivery systems should have a high absorption rate of the principal components and a fast rate of chemical release. Much research is conducted to meet these qualities. A polysaccharide hydrogel modified with carbon nanotubes was recently developed and analyzed by Bogdanova et al. (2020). The influence of carbon nanotubes on the polysaccharide matrix was observed through changes in the structural properties of the hydrogel, such as decreased pore size. It increased internal structure uniformity [108]. Hebeish et al. (2015) investigated the ability of starch nanoparticles to carry Indomethacin and Acyclovir active compounds, both of which are used to treat skin diseases and protect the skin from injury [112]. The critical factors for developing starch nanoparticle formulations, such as drug concentration, pore size, and active component release, were adjusted. For the Indomethacin formula, the ideal properties of starch nanoparticles were 0.5 g sodium tripolyphosphate with an active substance concentration of 20 g, and for Acyclovir, an optimal 0.5 g sodium tripolyphosphate with an active substance concentration of 50 g was established [112]. Reverse microemulsions and physical crosslinking by calcium ions for alginate-based nanoparticles and zinc ions for pectine-based nanoparticles were used to produce and characterise alginate and pectin polysaccharide hydrogel nanoparticles. The nanoparticles obtained had an average size of 100 nm, and the hydrogel concentration was 1010 particles/mL. 

Moreover, the nanoparticles produced were stable for up to two months, and their toxic activity against the human neuroblastoma cell line SH-SY5Y was negative [109]. Aerogels consisting of PEGs are being developed as innovative material formulations for biological applications in medicine. As a result, a biodegradable matrix of pectin aerogels containing maghemite nanoparticles was utilised to create two new material formulations, cylindrical monoliths and microspheres, with potential applications in targeted delivery and magnetic resonance imaging monitoring [110]. The material formulation was prepared using a mix of sol-gel and supercritical drying processes. Due to the presence of iron oxide nanoparticles, the magnetic characteristics of the resulting aerogels were expected. Furthermore, the iron oxide nanoparticles modified the colour of the aerogel from white to dark brown, increased the aerogel density by 38%, and increased the aerogel surface area by 15% [110]. Therefore, PEGs are finding more and more applications in the medical and pharmaceutical sectors due to their unique features such as hydrophilicity, elasticity, excellent biocompatibility, and improved biodegradability.

The release of medications with various physicochemical properties can be controlled and targeted using hydrogel particles made of polysaccharides. The effective encapsulation of numerous medications (including antibiotics, steroidal and non-steroidal anti-inflammatory drugs, etc.) might provide benefits by guaranteeing their targeted delivery and appropriate release.

The needs for wound healing were shown to be better met by polysaccharide-based hydrogels made of natural biopolymers, such as alginate, cellulose, chitosan, hyaluronic acid, etc., as compared to other hydrogels [21]. First, the polysaccharide polymer’s backbone has a lot of hydroxyl and carboxyl groups, which gives the hydrogel it forms a more effective water content and superior swelling performance, as well as unmatched moisturising qualities to absorb tissue exudate and barely detectable adherence to wound tissue [113]. The role of polysaccharide hydrogel in controlling healing is very complex, and its performance, as well as its suitability for clinical use and commercialisation, depend on its physicochemical qualities (e.g., mechanical, rheological, swelling, moisturising, and heat absorption properties) [113].

## 4. Health Benefits of PEGs

An outcome of a greener economy and a healthier diet is the adoption of biopolymers, such as PEGs in the production of edible food packaging, which besides protecting the shelf-life and improving the overall characteristics of foodstuff, are also able to confer beneficial health effects to consumers [114,115,116,117]. A multitude of research is based on the applicability of these polysaccharides-based biopolymers in the food industry [72,118,119,120], together with the incorporation of bioactive compounds derived from diverse by-products such as tomato [121,122,123], berries [124,125], apple, carrot, red-beet [126,127], and other vegetable and fruit sources [95,124,128,129].

**Figure 5:**
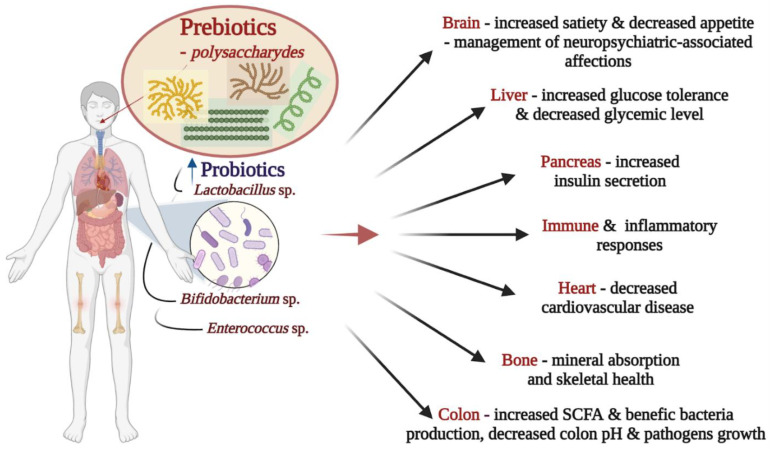
Prebiotic consumption and its possible impact on human health (image created using the BioRender application; https://app.biorender.com).

The compositional and functional disequilibrium of the gut ecosystem, known as dysbiosis, is accountable for the disclosure of several diseases from neurological affections [130,131], to the liver [132], cardiovascular [133], autoimmune diseases [134], food allergies [135], diabetes [136], obesity [137], cancer development [138], decreased calcium absorption [139], and even influence the severity of infectious disease such as COVID-19 [140,141]. The applicability of these compounds can exert an alleged “prebiotic” outcome on the health of humans and animals. Figure 5 shows the possible impact of prebiotics in human health [15]. However, several ongoing studies still exist to understand their specific effect on the gut and their beneficial after-effects [116,142,143]. This subchapter gives an overview of PEG benefits on gut microbiota, intestina microecology, and the “bridge effect” in obesity.

### 4.1. PEG Influences Gut Microbiota

Based on a recent perspective by Barratt et al. (2017), the production of ‘‘microbiota- directed’’ foods is an essential step to improve and establish technologies that create reasonably priced foods that encourages well-being [83,144]. To sustain the gut microbiota, these foods should be manufactured based on constituents that have a “prebiotic” effect [144]. Gibson and Roberfroid first conceived this “prebiotic” notion in 1995 [145]. It encompasses non-digestible elements which are easily fermented by the colon ecosystem and also have the positive effect of encouraging the activity and growth of some particular gut microorganisms, being advantageous to the physical function of the gastrointestinal tract [116]. As the world consumes multiple forms of carbohydrates, these can be used by the commensal microbiota, and polysaccharides are mainly assimilated in the colon, especially by the beneficial probiotic bacterias from the *Lactobacillus* sp. and *Bifidobacteria* sp. [146].

Martău et al. (2019) categorise the most applied PEGs as alginate, carrageenans, cellulose derivatives, chitosan, gums, pectin, and starch [118]. These polysaccharides are mainly extracted from natural sources, such as pectin from apple pomace, bamboo shoots, citrus by-products, and many other fields [82,147]. A recent study by Tingirikari (2019) shows that pectic polysaccharides, such as arabinan, arabinogalactan, rhamnogalactouronan, and polygalacturonic acid are just gradually fermented. Consequently, by attaining the colon’s more distant region, they supported the growth of *Bacteriodes* and *Bifidobacteria* species. They made a substantial amount of short-chain fatty acids (SCFA), especially lactate propionate and acetate [148]. These metabolites are important in conserving a healthy gut and preventing the formation of several intestinal disorders [146]. Pectin can also be used as a hydrocolloid in producing foods for adults with oropharyngeal dysphagia, a complex swallowing disorder. According to estimates, this disease impacts 8% of people worldwide and has several after effects. An essential element is altering the consumed food liquids density by making swallowing smoother. Thus, based on several studies, pectin can be used as a prebiotic effect as a thickener in various food matrices [149,150].

On the other hand, starch and chitin are also mainly digested in the special section of the gut. Starch is found in many natural plants such as staple foods, potatoes, and bananas, employed for energy storage and is composed mainly of amylopectin and amylose [151]. The primary metabolite of resistant starch is butyrate [152], which promotes the growth of Firmicutes species and some species from *Bifidobacterium*, *Bacteroides*, *Eubacterium* and *Ruminococcus* [153]. Chitin, after cellulose, is the next most valuable long-chain polymer extracted from natural sources, such as mushrooms and algae or from non-vegetable sources such as the exoskeleton of crabs, shrimps, and insects, ore even in the cell walls of fungi [154,155,156]. The derivatives from chitin are comprised of a wide range of β-1,4-glycosidic bonds, which can also resist gut digestion and promote the development of the *Lactobacillus sp.* and of *Bifidobacterium bifidium*, and decrease the growth of *Proteobacteria* [157]. Based on various studies, they also promote the production of butyrate and propionate metabolites [157].

### 4.2. PEG Influences on Intestinal Microecology

The term “intestinal microecology” refers to the three components of the intestinal mucosal barrier: intestinal microbiota, mucosal immune system, and the intestinal epithelial cells [25,26,27,158]. Due to dietary fluctuations, gastrointestinal dysbiosis is widely believed to have a significant role in the progress of metabolic disorders such as obesity, chronic inflammation, and insulin resistance. The intestinal microbiota has the principal role in the intestinal microenvironment. Dietary PEGs modulate the gut microbial population by reducing pathogen development, controlling commensal bacteria and probiotics, and increasing host-microbial interactions, resulting in health benefits [25,26,27]. For example, in vivo, the polysaccharides gels extracted from *Polygonatum kingianum* (0.1 mg/kg) optimised the intestinal microecology by reducing the number of *Bacteroidetes* (3.31%) and *Proteobacteria* (31.19%) and increasing the number of *Firmicutes* (23.15%) [27]. Moreover, with a higher dosage (800 mg/kg) of *Maydis stigma* polysaccharide, the abundance of *Lactobacillus* and *Bacteroides* significantly were improved, highlighting their prebiotic properties [25]. The study of Tao et al. (2017) revealed a positive correlation between protective bacteria such as *Lactobacillus, Bifidobacterium* (probiotics), *Butyricicoccus*, *Clostridium*, *Lachnospiraceae*, *Rikenellaceae* and the anti-inflammatory cytokines (e.g., IL-4, IL-10, and IL-11) [158].

Several strategies have been proposed to produce more stable PEGs in the gastrointestinal tract [159,160]. These include coating the pectin gel with hydroxypropyl methylcellulose and changing the structure of pectin granules at the isolating stage to get different permeability and biodegradability characteristics in the intestine [160]. Drug release was lowest (2 h, 23.40%) at pH 1.2, moderate at pH 6.8 (4 h, 25.88%) and highest (4 h, 70.20%) at pH 7.4 [159]. Using whey protein isolate polysaccharide hydrogels, researchers evaluated in vitro digesting properties. For the release of black carrot concentrate, whey protein was coupled with pectin, gum tragacanth, and xanthan gum in this research. Black carrot concentrate was successfully released into the simulated intestinal fluid at a rate of 95% using whey protein-pectin hydrogels and only 73.43% using whey protein-xanthan gum hydrogels [161]. The most challenging biological role of PEGs is in mucosal immune system enhancement. Inhibiting the development and spread of cancer cells, avoiding bacterial infection, decreasing blood cholesterol levels, and mending wounds are functions attributed to the biological activity of polysaccharides. Chrysanthemum polysaccharides (50 mg/kg) can potentially alleviate 2, 4, 6-trinitrobenzene sulfonic acid-induced colitis by repairing the immune system and promoting the establishment of a healthy intestinal microecology [158]. Furthermore, gum acacia polysaccharide aerogel has been administered to treat intestinal inflammation and cover inflammatory areas. It has been found with antibacterial and antioxidant properties [162].

PEGs were suggested as a possible modulator for inflammatory factors such as tumour necrosis factor α (TNF α), nuclear factor kappa B, and interleukin (IL-1, IL-6) [25,26,159,162,163]. Specifically, polysaccharide gels isolated from *Moringa oleifera* leaf (MO) positively influence gut microecology by their prebiotic effect. To examine the influence of MO (10%) on intestinal integrity, serum endotoxin, TNF-α, diamine oxidase, and D-lactate levels were measured. These were all shown to be decreased by 18%, 32%, and 24%, respectively. In addition, villus height and mucosal thickness increased (*p* < 0.1), demonstrating an improvement in intestinal tissue integrity in the MO group [26]. In another study, a pectin/zein-based hydrogel with *Lactobacillus rhamnosus* GG-derived protein (p40) was made to transactivation the epidermal growth factor receptor in intestinal epithelial cells and prevent the intestinal epithelium from inflammation and injury. This approach improved mice’s postnatal bodyweight growth and intestinal performance by delivering bioactive compounds to the small intestine and colon (day 2 to 21). Similar to humans, neonatal p40 therapy protected adult mice against intestinal damage and colitis by boosting the development of protective immune responses [164]. The effects on ulcerative colitis of konjac gum, xanthan gum, glycerol, and sodium alginate hydrogel were investigated. In vivo data showed that this hydrogel was an effective medication for ulcerative colitis, nontoxic, and decreased the spleen and thymus index [159]. Luo et al. (2019) results showed that the quantities of IL-6 and TNF-α in the cell supernatant were considerably reduced (*p* < 0.01) following administration of *Bletilla striata* polysaccharide (BSP) due to their lower intestinal epithelial cell permeability. BSP treatment also preserved zonula occludens and occludin protein expression in intestinal epithelial cell injured lipopolysaccharide [163]. Su et al. (2018) evaluated graphene-alginate associations. After having the surface of few-layer graphene modified with alginate, the systemic and intestinal levels of few-layer graphene were significantly raised, whereas intestinal epithelial cell internalization was dramatically decreased [165]. In vitro studies indicated that a pectic polysaccharide was obtained from water decoction of Xinjiang *L. brabarum* using anion exchange chromatography and gel filtration. This may minimise endoplasmic reticulum stress and the unfolded proteins response. It could also protect porcine jejunum epithelial cell line cells from apoptosis stimulated by the endoplasmic reticulum stress [166]. Thus, PEGs might be excellent candidates to enhance the stability of intestinal microecology.

### 4.3. PEG Influences as “Bridge Effect” in Obesity

Obesity is a significant health problem that has increased over the last decade and has significantly contributed to the burden of non-communicable diseases, such as type 2 diabetes, cardiovascular disease, hypertension, and cancer. People’s quality of life is also impacted by mechanical problems brought on by increased weight, such as osteoarthritis and sleep apnea. One of the leading causes of the obesity epidemic is the globalization of food systems, which produces more processed and affordable food and encourages passive overconsumption of low-density, high-energy food products. Another vital factor which has significance on obesity is the decreasing trend of physical activity practice [167,168]

The correlation between PEGs and the health benefits is a variant that supports and improves the factors that lead to or sustain a specific pathology, in this case, obesity. One of the main factors that can help enhance obesity is their role as a carrier for bioactive compounds. Delivery setups have been developed to prevent the deterioration of sensitive chemical components during the food production processes, as well as enzymes for digestion and harsh environmental conditions and area-specific gastro-intestinal transportation and controlled release of hydrophilic and hydrophobic nutraceuticals [19]. Lipid-dependent networks, biopolymers, and systems inspired by nature, such as PEGs, are the most used types of delivery methods. Protein-based hydrogels are one of these approaches that are particularly praised by many areas for their outstanding properties, such as maximum dietary advantages, excellent functional qualities, amphiphilic character, biocompatibility, and biodegradability. Polymeric chain cross-linking prevents the matrix from disintegrating and upholds the mechanical stability [19,169].

Regarding the ability and capacity of polysaccharide-based edible substances to act in obesity, some studies prove their role compared with other kinds of meds used in this pathology [170]. A review conducted by Guarino et al. (2022) synthesized randomised clinical studies and one retrospective clinical study on obese children and adults, with or without metabolic syndrome. All studies are concentrated on one product called Policaptil Gel Retard (PGR). The PGR complex is made of processed raw materials such as glucomannan (from *Amorphophallus konjac*), cellulose (from *Opuntia ficus-indica*), chicory root (Cichorium intybus), and mucilage (from *Althaea oficinalis*, *Linum usitatissimum*, and *Tilia platyphyllos Scop*) as well as various types of dietary fiber. The results of PGR are promising even if the number of studies is still low. The effect in children was a decreased appetite, a decreasing level of postprandial triglycerides, and peak postprandial blood glucose levels in adults. Adults who received PGR for 30 days and followed a normocaloric diet had decreased lipid levels. As a long-term treatment, PGR reduced body mass index and waist circumference, improved insulin sensitivity, and improved the circulating lipid profile [171].

Therefore,—PEG as carriers of bioactive compounds—can play a role in the obesity crisis [169]. From the point of view of food or food products consumption, the source of bioactive compounds can still show a susceptibility from the production until ingestion, through the passage of the gastro-intestinal tract [28]. To improve the stability of the food, more precisely of bioactive compounds, encapsulation in different food hydrogels can preserve their action in the human body. However, there are still some questions about the interactions of gels’ mechanisms and other nutrients. Regarding their benefits, the comprehensive understanding is an open field for further research to better design food with specific effects in various pathologies. Therefore, more studies need to be conducted on the interactions of PEGs and foods and further on the interaction with the human body [18,19].

## 5. Conclusions

The compatibility between bigels, serving as vehicles for both hydrophilic and lipophilic bioactive molecules, and personalised 3D food printing might have impactful results regarding the prevention or the treatment of individual needs in humans. However, in vitro and in vivo studies would be desirable to investigate the bioavailability and bioaccessibility of the active compounds during digestion.

PEGs play a significant role in developing smart materials, both as ingredients and as edible, biodegradable, and safe coating materials. Methods and techniques for providing and exploring them are necessary. They are finding more and more applications in the medical and pharmaceutical sectors due to their unique features such as hydrophilicity, elasticity, excellent biocompatibility, and improved biodegradability.

Dietary PEGs modulate the gut microbial population by reducing pathogen development, controlling commensal bacteria and probiotics, and increasing host-microbial interactions, resulting in health benefits. The role of PEGs as carriers of bioactive compounds has a demonstrated health effect on obesity.

As PEGs are organic composites derived from natural sources, they may have a variety of applications in both food and medical/pharmaceutical sectors, as well as for developing biodegradable materials with positive environmental impact. Still, these valuable products must be more investigated to identify and understand all the physical, chemical, and biological interactions among them and other organic and inorganic structures.

## Figures and Tables

**Table 1:** Main characteristics of aerogels, cryogels and xerogels, respectively.

Characterization	Polysaccharide	Aerogel	Cryogel	Xerogel	Reference
Bulk density (g/cm^3^)	Pectin	~0.083	~0.073	~1.057	[9]
	Pectin/TiO_2_	0.11–0.24	N/A	N/A	[67]
	Chitosan	0.07–0.26	0.03–0.12	~1.30	[62]
	Chitosan/xantan gum	0.24–0.36	0.099	N/A	[68]
Volume shrinkage (%)	Pectin	~30	f10–13	90	[9]
	Chitosan	~20	~0	~80	[62]
	Cellulose	~8.5–19.2	~41	~89–93.4	[29]
Porosity (%)	Pectin	~94	~95	~30	[9]
	Chitosan	80–95	90–98	~10	[62]
	Cellulose	~92.7–96	94.3	70.2–80.3	[29]
	Chitosan/xantan gum	~60–68	~85		[68]
BET specific surface area (m^2^/g)	Pectin	~362	10–20	N/A	[9]
	Pectin/TiO_2_	339–461	N/A	N/A	[67]
	Chitosan	200–270	50–70	N/A	[62]
	Chitosan/xantan gum	~5.7–17.5	~9.1	N/A	[68]
	Cellulose	287–303	23	0.81–107	[29]
	Corn starch	~64	N/A	N/A	[69]
Network morphological aspect (according to scanning electron microscopy, SEM)	Pectin	Mesopores and small macropores (50–150 nm in diameter)	Large macropores (0.5–5 µm in diameter)	Dense network	[9]
	Pectin/TiO_2_	Macropores >50 nm Mesopores (2–50 nm) Micropores (<2 nm)	N/A	N/A	[67]
	Chitosan	Size of pores of about ~0.25 µm	Largest pores (~0.5 µm)	Absence of pores	[62]
	Cellulose	Mezopore size distribution between 5–200 nm	Nanofibrils between 50–100 nm	Macropores size between 50–120 µm	[29]
	Chitosan/xantan gum	Dense and smooth surface	Granular-like structure and rough surface	N/A	[68]

TiO_2_—titanium dioxide, BET—Brunauer, Emmett et Teller method, N/A—not determined. Adapted according to [9,29,62,67,68,69].

## Data Availability

Not applicable.
